# The Use of Multiple Primary Outcomes in Randomized Controlled Trials of Chinese Herbal Medicine

**DOI:** 10.1155/2021/9975351

**Published:** 2021-04-21

**Authors:** Jing Hu, Shuo Feng, Xiaoli Zhang, Huina Zhang, Yanxiang Ha, Chongyang Wei, Xuejiao Wang, Rui Zhang, Xing Liao, Bo Li

**Affiliations:** ^1^Department of Clinical Epidemiology, Beijing Traditional Chinese Medicine Hospital, Capital Medical University, Beijing Institute of Traditional Chinese Medicine, Beijing 100010, China; ^2^Editorial Office of Medical Journal of Chinese PLA, Beijing 100842, China; ^3^Center for Evidence Based Chinese Medicine, Institute of Basic Research in Clinical Medicine, China Academy of Chinese Medical Sciences, Beijing 100700, China

## Abstract

**Background:**

Multiple primary outcomes are commonly used in randomized controlled trials (RCTs) of Chinese herbal medicine (CHM). Analysis and interpretation of the results of CHM RCTs with many outcomes are not clear. No previous studies have systematically assessed the use of multiple primary outcomes in this area. This study aimed to assess the reporting of multiple primary outcomes and the statistical methods used to adjust multiplicity in RCTs of CHM.

**Methods:**

Search for RCTs of CHM published in English between January 2010 and December 2019 in MEDLINE, EMBASE, and the Cochrane Central Register of Controlled Trials (CENTRAL) was undertaken. We randomly selected 20% of the included RCTs as the analyzing sample of this study. The number of multiple primary outcomes, the methods used to adjust the multiplicity in statistical analysis and sample size estimate, and the trial information were collected. For RCTs that adopted multiple primary outcomes without the multiplicity adjustment, we used Bonferroni correction to adjust.

**Results:**

227 CHM RCTs were included in our study. 92 (40.5%) failed to report what their primary outcome was. Of 135 (59.5%) RCTs that reported primary outcome, 93 (68.9%) reported one and 42 (31.1%) reported more than one primary outcome (range 2–5). Of 42 RCTs that reported multiple primary outcomes, only 5 adjusted for multiple outcomes. If multiplicity had been accounted for using Bonferroni correction, 10 (37.0%) RCTs that reported a significant result had demonstrated a nonsignificant result, giving the adjusted *P* value. Only one of the 42 RCTs calculated sample size based on multiple primary outcomes. Adopting multiple primary outcomes showed a slow growth trend with the publication year. The proportion of primary outcome reported explicitly in RCTs was different in terms of the nationality of the first author (*P*=0.004), in which mainland China has the lowest proportion (55.8%). The highest percentage of the studies with primary outcome reporting explicitation was mental and behavioural disorders (83.3%), and the most frequently adopting multiple primary outcomes were studies on the disease of the nervous system (66.7%). The percentage of reporting primary outcome explicitly was associated with sample size (*P* < 0.001); for the percentage of RCTs adopting multiple primary outcomes, there was no statistically significant difference (*P*=0.739).

**Conclusions:**

Multiple primary outcomes are prevalent in CHM RCTs. However, appropriate methods are not usually taken in most of the analyses to safeguard the inferences against multiplicity. Sample size estimation based on multiple primary outcomes is still lacking. These issues complicate the interpretability of trial results and can lead to spurious conclusions. Guidelines to improve analyzing and reporting for multiple primary outcomes in CHM RCTs are warranted.

## 1. Introduction

Chinese herbal medicine (CHM) alone, or in combination with Western medicine (WM), has been widely used for patients with different diseases in mainland China [1–4]. Since the first randomized controlled trial (RCT) of CHM was published in 1982 [5], RCTs have been widely used to assess the clinical efficacy of CHM [6]. Although ICH Harmonised Tripartite Guideline Statistical Principles Clinical Trials E9 (ICH E9) recommends RCT designed with a single primary outcome [7], the effect of interventions is always multidimensional. A single outcome is insufficient to describe all the effects of an intervention on a complex disease in RCTs. However, multiple health outcomes may need to be investigated to assess all the relevant aspects of the disease. These multiple health outcomes are often correlated, especially for this efficacy on both physical and psychological outcomes. Then, multiple primary outcomes are often incorporated in RCTs due to interest in characterizing how a treatment influences a range of responses [8]. CHM, namely, Chinese herbal formulas, are composed of ingredients chosen to function in combination with each other and are particularly reflective of this practice. In WM, medications are usually prescribed individually for a specific effect. In Chinese herbal formulas, each herb has a different role to help the human body achieve harmony [9]. Therefore, reporting more than one primary outcome in CHM trials may be appropriate because a single measure may not sufficiently characterize the effect of a Chinese herbal formula on a broad set of domains [10, 11]. Multidimensional primary outcomes, which can incorporate the laboratory test, traditional Chinese medicine- (TCM-) diagnosed information (e.g., tongue coat, pulse, face color, and mind), and clinician-concerned and patient-reported outcomes have been proposed [12, 13]. When there is a lack of clear consensus on the most important clinical outcome, combined with the need to examine clinical effectiveness on related outcomes spanning disparate domains, encourage the use of multiple primary outcomes [14].

Normally, researchers often specify an outcome to serve as the primary one, with some other outcomes listed as secondary to adhere to the statistical design principle. While it is common to collect and report multiple primary measures in practice, the appropriate and efficient analysis for multiple primary outcomes is not fully established [14–16]. Choosing an appropriate method for dealing with multiple primary outcomes is important because clinical interpretations can be difficult for those multiple conflicting results.

There are mainly four kinds of approaches accounting for multiple outcomes that have been proposed, assessed, and reviewed [15]. The most common method for analyzing multiple primary outcomes is separate testing of each individual outcome, sometimes with but most often without adjustment for multiple testing [16, 17]. In terms of statistical principle, this method increases the probability of making at least one false significant result, and this could lead to an erroneous conclusion [18]. The second method is controlling the Type I error for multiplicity and the most common technique observed was the Bonferroni adjustment [19]. The third approach involves combining the multiple outcomes into a single (composite) outcome and performing a single test [20]. The fourth method uses global testing using simultaneous (joint) tests [21].

Furthermore, the sample size estimation is an important part of designing RCT. The number of primary outcomes and the correlations among them should be considered when estimating the sample size, which, if optimal, could help to ensure that the trial is efficient, ethical, and cost-effective. For trials with a single primary outcome, the sample size estimation is often univocal. While for trials with multiple primary outcomes, these outcomes and the correlations among them should be prioritized before the sample size estimation [22, 23].

We assessed the prevalence of reporting and adopting multiple primary outcomes in RCTs of CHM. CHM RCTs were chosen because they have a profound social and economic cost and are the focus of a number of prevention and intervention trials. The use of multiple primary outcomes in CHM RCTs is particularly common because efficacy mechanism complexity is multifaceted. Clinicians may be interested in the impact of a CHM on different aspects. For RCTs that reported multiple primary outcomes but without the multiplicity adjustment, we used Bonferroni correction to adjust.

## 2. Materials and Methods

We conducted the current study, which focused on CHM trials published in English databases from 2010 to 2019. Given the large number of the published studies, we randomly selected 20% of them. We aimed to describe the following: (1) the prevalence of RCTs reported primary outcome, (2) the prevalence of RCTs that adopted multiple primary outcomes, (3) the percentage of multiple adjustment for the multiple primary outcomes in the process of statistical analysis and sample size estimation, and (4) factors distributed in primary outcome reporting explicitation and adopting multiple primary outcomes.

We present the following article in accordance with the PRISMA (Preferred Reporting Items for Systematic Reviews and Meta-Analyses) checklist.

### 2.1. Search Strategy

MEDLINE, EMBASE, and CENTRAL (Cochrane Central Register of Controlled Trials) were searched by JH, and only RCTs published in English between 2010 and 2019 were selected. Medline was used to obtain articles that matched “clinical trials” and included the keywords “chinese herbal medicine” or “traditional Chinese medicine”. The detailed MEDLINE search strategy is available in Supplementary Materials.

### 2.2. Eligibility Criteria

RCTs published in English language were selected if they were parallel, crossover, factorial and N-of-1 trials, and studying oral CHM alone or in combination with other interventions, with different preparation forms (e.g., oral liquid, tablet, capsule, pill, granule, and decoction). There is no limitation on diseases. All the following were excluded: (1) phase I or pharmacokinetics trials, (2) for healthy subjects, (3) self-described preliminary or pilot studies, (4) follow-up or secondary analysis of the original data, and (5) protocols or conference paper.

In addition, RCTs were excluded if the studies focused on nontraditional Chinese herbs; plant extract product is also excluded because it is approved as a nonherbal product by China's Food and Drug Administration (FDA) and it belongs to the same category as WM, which is out of the rules of TCM.

### 2.3. Selection of Studies

Firstly, we imported 39,116 related records into the reference manager software and built a database. Secondly, we used the random sampling method used in other studies [16, 24, 25] to select target samples for analysis. SAS for Windows (version 9.4; Order Number: 9C1XJD) was used to generate a 20% random sampling number table and 7,824 records were selected. We numbered and sorted the selected records. Thirdly, four reviewers (YXH, XJW, RZ, and CYW), divided into two groups (in pairs), individually and independently screened the titles and abstracts of the selected studies to determine those potentially met the inclusion criteria and 475 related trials were found. Finally, we then obtained the full text of these trials and independently reviewed to find the exact trials that met the inclusion criteria; 227 trials were picked out. Any inconsistency during this process was resolved by discussion with a third party (JH and XL).

### 2.4. Data Extraction

For each RCT, the results in the abstract and the methods used for sample size estimation and statistical analysis were examined. The numbers of primary outcomes, secondary outcomes, and methods (if any) used to account for multiple primary outcomes were extracted. An outcome was identified as primary if it was explicitly stated in the abstract, methods, results, or tables or if it was clearly implied in the aims of the RCT. We also considered the outcome as primary outcome if it had been explicitly referenced in the sample size estimation. Other outcomes were extracted as secondary outcomes. Side effects and adverse events were not extracted. In addition, publication details (e.g., year, authors, and journal), participants, disease (coded by the International Classification of Disease revision 10 (ICD-10)), interventions, sample size, and sample size estimation were also extracted.

### 2.5. Data Analysis

Firstly, we performed a descriptive statistical analysis for all the extracted information of the included RCTs. For RCTs that reported multiple primary outcomes but without the multiplicity adjustment, we used Bonferroni correction to adjust, which is based on the probability of obtaining a false positive. It is a method where the significance level is divided by the number of primary outcomes and then compares each single outcome's *P* value with the adjusted level of *a*/*K* rather than *a*, where *K* is the total number of primary outcomes.

Factors distributed in primary outcome reporting explicitation and adopting multiple primary outcomes, including countries and sample size, were examined by chi-square test. A *P* value of 0.05 was used to assess statistical significance. Analyses were performed using SAS for Windows (version 9.4; Order Number: 9C1XJD).

## 3. Results

### 3.1. Screening of Included Studies

**Figure 1:**
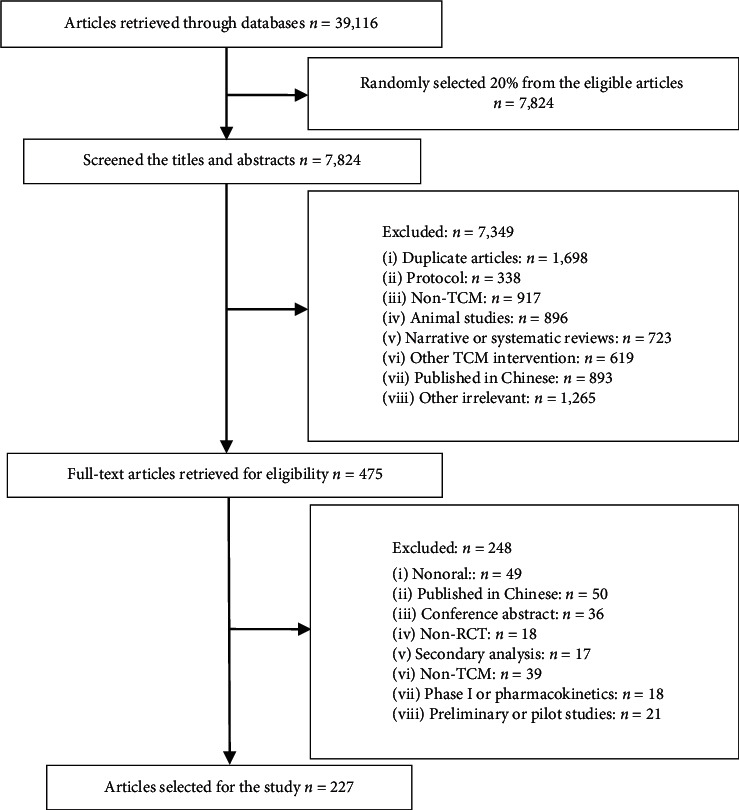
Flow chart of study selection.

We identified and selected 227 RCTs of CHM that met the inclusion criteria. Details of the study screening process can be seen in Figure 1.

### 3.2. Basic Characteristics of Included RCTs

Of the 227 CHM RCTs, 197 (86.8%) were conducted from mainland China, and 193 (85.0%) were designed with two arms, 28 (12.3%) with three arms, and 6 (2.6%) with four arms. The sample size ranged from 12 to 3,143 participants (median: 115, quartile range [IQR] 72–228).  Table 1 summarizes the characteristics of these trials.

### 3.3. Primary Outcomes and Adjustment

**Figure 2:**
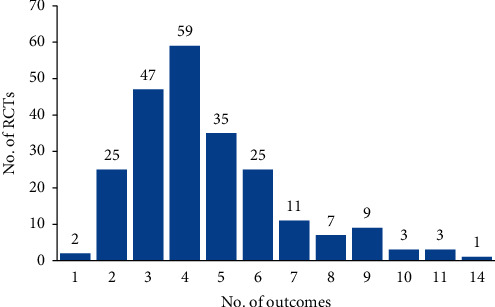
Number of reported outcomes in 227 included RCTs.

The median number of outcomes was 4 (IQR 3 to 6, range 1–14) in 227 CHM RCTs (Figure 2). Of the 227 RCTs, 92 (40.5%) did not clearly specify any primary or secondary outcome, 93 (68.9%) explicitly reported a single primary outcome, 42 (31.1%) reported multiple primary outcomes (in which 24 RCTs had 2 outcomes, 12 had 3 outcomes, 5 had 4 outcomes, and 1 had 5 outcomes).

Of the 42 RCTs with multiple primary outcomes, only 5 (11.9%) had adjusted for multiple primary outcomes, in which three of them used Bonferroni correction and two used Benjamini–Hochberg adjustment. Of the remaining 37 RCTs, ten of them reported “*P* < 0.05” in the full text instead of the actual *P* value. Then, we used Bonferroni's adjustment to account for the multiplicity in the other 27 RCTs with *P* value. Of the 27 RCTs, ten (37.0%) that reported an effective intervention would have drawn different conclusions giving the adjusted *P* value.

### 3.4. Sample Size Estimation

Sixteen (38.1%) of the 42 trials that reported multiple primary outcomes did not report the process of estimating sample size. Twenty-five of the trials reported sample size estimation based on one outcome. Only one RCT reported sample size estimation that involved more than one primary outcome [26]. This study adopted 3 primary outcomes, 3 sample sizes of these outcomes were estimated with a total significance level of 5% according to Bonferroni correction of the *P* value (*P* < 0.017), and then the largest value was selected for the final sample size.

### 3.5. Viewing the Results by Publication Year and Countries

**Figure 3:**
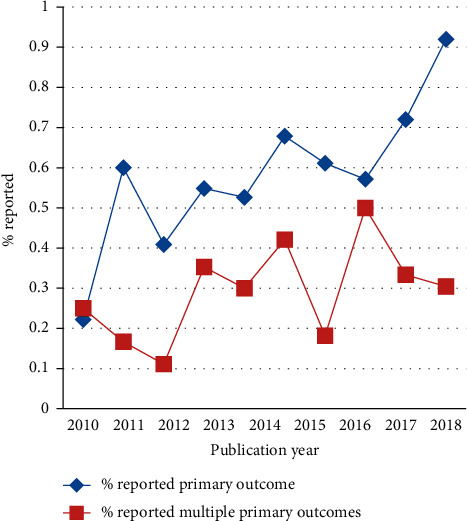
Percentage of RCTs that reported primary outcome and primary multiple outcomes by publication year.

In general, the percentage of primary outcome reported explicitly was increasing by year between 2010 and 2019, from 22.2% in 2010 to 92.0% in 2019. Adopting multiple primary outcomes showed a slow growth trend with the publication year (Figure 3). The proportion of primary outcome reported explicitly in RCTs was different in terms of the nationality of the first author (*P*=0.004; see Table 2), in which mainland China has the lowest proportion (55.8%).

### 3.6. Viewing the Results by Disease Area

According to ICD-10 classification, the highest prevalence of the included RCTs focused on circulatory disease (*n* = 36), followed by the genitourinary system (*n* = 28) and digestive system (*n* = 27).

The highest percentage of the studies with primary outcome reporting explicitation was mental and behavioural disorders (83.3%), followed by diseases of the respiratory system (80.9%); diseases of the skin and subcutaneous tissue (75.0%); and symptoms, signs, and abnormal clinical and laboratory findings, not elsewhere classified (75.0%).

The most frequently adopting multiple primary outcomes were studies on the disease of the nervous system (66.7%), followed by mental and behavioural disorders (60.0%) and certain infectious and parasitic diseases (50.0%; see Table 3).

### 3.7. Viewing the Results by the Sample Size

Based on the quartiles, the sample size could be divided into three levels of small (sample size <72), medium (72 to 227), and large (≥228). The percentage of reporting primary outcome explicitly was associated with sample size (*P* < 0.001). For the percentage of RCTs adopting multiple primary outcomes, there was no statistically significant difference (*P*=0.739; see Table 4).

## 4. Discussion

We randomly selected 227 RCTs published in English between 2010 and 2019 and analyzed the consistency in the reporting and analysis of multiple primary outcomes in CHM. Among the representative, 40.5% did not clearly specify any primary outcome. This suggested the reporting of primary outcome explicitly is relatively low in trials of CHM. Failure to reporting primary outcomes may lead to selective outcome reporting [27]. The International Standards for Clinical Trials Registries established by the World Health Organization has stated that both the primary and secondary outcomes should be defined and prespecified [28]. CONSORT statement also claimed that primary outcomes should be clearly and explicitly stated in all peer-reviewed published RCTs [29]. Our study demonstrated that the specification and explicitation of the primary outcome in clinical trials of CHM need to be improved. Inexplicit primary outcome reporting also has been reported in some previous studies in pediatrics, depression, neurology, and psychiatry research areas [14–17]. The percentage of reporting primary outcome in our study was generally lower than these studies, although the percentage had an upward trend with publication year.

In our study, nearly one-third (31.1%) of included RCTs adopted multiple primary outcomes, while only 5 of these 42 RCTs adjusted for the multiplicity. For the statistical analysis, separate testing of each individual primary outcome, without adjustment for multiple testing, was the most commonly used method to deal with the multiplicity in currently published CHM trials.

A familiar drawback of this approach is the probability of obtaining statistically significant results due to the chance may increase [14, 18]. Practically, what we were concerned about is that it can be falsely concluded that a treatment has significant benefits when the results are actually due to chance, rather than to treatment efficacy (Type I error). When multiple outcomes are analyzed without any adjustments, the Type I error would increase. In our study, for the trials that did not account for multiplicity, we used Bonferroni correction and found that 10 (37.0%) that reported an effective intervention would lead to false positive conclusions. That implied the control of Type I error rate for the multiple primary outcomes is critical.

There are a variety of statistical adjustment methods that can be used to control the Type I error for multiplicity [30]. In particular, the *P* value-based approaches are the most commonly used. These approaches can be classified into two types: single-step and multistep procedures. The Bonferroni method is a single-step procedure that is usually recommended because of its simplicity and broad applicability [19], even though it was considered to be conservative when the outcomes are positively correlated [31]. Holm procedure is a multistep, step-down procedure [32], while the Hochberg procedure is step-up [33], which are useful for outcomes with any degree of correlation.

Some other statistical analysis methods can also be used to multiple primary endpoints without the need to adjust *P* values. A comprehensive evaluation method can combine the multiple outcomes into a single (composite) outcome, using a variety of pooling rules or scoring algorithms, such as taking a simple average of the outcomes or using conjunctive or compensatory rules, and then test treatment difference on this composite outcome [20]. The global statistical test can provide a univariate test statistic to describe overall benefit and respect the correlated nature of the multiple outcomes instead of multiple statistical tests [21]; this approach is useful to test a treatment's global benefit based on multiple outcomes [34].

Since the holism perspective of TCM, as well as the multidimensional of the reported outcomes (patient-reported, laboratory test, clinician-rated and TCM syndrome outcomes, etc.), it is not practical to identify a single most important outcome as the primary outcome to summarize the effect of CHM [35]. Our previous study also had proposed an efficacy evaluation system with multiple primary outcomes, which is based on the holism benefit of TCM, integrated the primary outcome by three domains: western medicine-specific outcome, TCM syndrome outcome, and quality of life [12].

Determine the sample size that guarantees the prespecified power is an important task in the design phase of clinical trials, and the sample size estimation should be based on the primary outcomes. When a single primary outcome is used, the estimate of sample size has been well studied [36] while when estimating the sample size for trials with multiple primary outcomes, these outcomes and the correlations among them should be considered [15, 22, 23].

For the included studies that adopted multiple primary outcomes, only one RCT estimated sample size based on multiple primary outcomes. Others just used one primary outcome to estimate sample size, while this maybe causes insufficient power to find statistically significant results. The simple and most commonly used adjustment method is using a multiplicity-adjusted significance level within the estimate, estimating for all the primary outcomes, and then selecting the largest sample [37].

In order to help improve practice in this area, we suggest that all CHM RCTs report the following:

- The authors should clearly specify a single primary outcome of the trial or multiple primary outcomes along with a strategy to account for multiplicity
- The authors should consider the use of more principled methods to minimize the chance of spurious results due to multiplicity by accounting for multiple primary outcomes
- The authors should report the sample size estimation and use all primary outcomes with a multiplicity-adjusted significance level in the estimation for multiple primary outcomes RCTs
- The authors should specify a limited number of secondary outcomes, along with a justification for their inclusion
- The authors should adopt the CONSORT guidelines, the current ICH guidelines, and other related standards or act, which could help improve the timely dissemination and appropriate interpretation of results from clinical trials

## 5. Strengths and Limitations

To our knowledge, this is the first study to present an overview of multiple primary outcomes adopting and adjustment in CHM RCTs. We chose to assess a random 20% sample as we believe this represents a comprehensive and feasible sample. We focused on studies published in English because those RCTs are believed as having higher methodological quality and more rigorous publication standards than those published in Chinese [38, 39]. Hence, if a significant problem exists in this group, then our findings will likely underestimate the extent of the problem in all CMH RCTs.

This study also has some other limitations. The included studies compromised both confirmatory and exploratory clinical trials, whereas, for explanatory trials, the major objective of which is to frame future research or explore new hypotheses, the multiplicity adjustment consideration is less important. As a comparative effect design, rigorous multiplicity adjustment and Type I error control in exploratory trials may lead to difficulty in achieving the major objectives. Thus, the finding in our study may be potentially exaggerated. Therefore, additional research on a wider scope and specific types of design is needed to furtherly assess the multiplicity adjustment in CHM.

## 6. Conclusions

From the selected sample of randomized controlled trials on Chinese herbal medicine, this study demonstrated that the primary outcome reporting was generally inexplicit. Multiple primary outcomes were commonly adopted while the multiplicity adjustment was rarely addressed. An appropriate statistical method for analysis and sample size calculation to safeguard the inferences against multiplicity should be used.

## Figures and Tables

**Table 1:** Summary of the characteristics of included RCTs.

Variable		Number of RCTs (*n* = 227)	Variable		Number of RCTs (*n* = 227)
*Year*	2010	18 (7.9%)	*Country*	Mainland China	197 (86.8%)
	2011	20 (8.8%)		Hong Kong	11 (4.8%)
	2012	22 (9.7%)		Taiwan	8 (3.5%)
	2013	31 (13.6%)		Singapore	2 (0.9%)
	2014	19 (8.4%)		America	2 (0.9%)
	2015	28 (12.3%)		Australia	2 (0.9%)
	2016	18 (7.9%)		Brazil	1 (0.4%)
	2017	21 (9.2%)		India	1 (0.4%)
	2018	25 (11.0%)		Iran	1 (0.4%)
	2019	25 (11.0%)		Netherlands	1 (0.4%)

*Arms per trial*	2	193 (85.0%)	*Sample size*	Korea	1 (0.4%)
	3	28 (12.3%)		Range	12–3,143
	6	6 (2.6%)		Median (IQR)	115 (72–228)

**Table 2:** The primary outcome reporting percentage with the first author's country.

Country of first author	No. of RCTs	No. of RCTs that reported primary outcome	No. of RCTs that adopted multiple primary outcomes
Mainland China	197	100 (55.8%)	37 (33.6%)
Hong Kong	11	10 (90.9%)	1 (10.0%)
Taiwan	8	6 (75.0%)	2 (33.3%)
Other countries	11	9 (81.8%)	2 (22.2%)
Total	227	135 (59.5%)	42 (31.1%)
*P* value		0.004	0.239

**Table 3:** Disease classification (ICD-10) of RCTs reporting primary outcomes and multiple primary outcomes.

Disease classification (ICD-10)	No. of RCTs	No. of RCTs that reported primary outcome	No. of RCTs that adopted multiple primary outcomes
Certain infectious and parasitic diseases	8	2 (25.0%)	1 (50.0%)
Neoplasms	19	9 (47.4%)	1 (11.1%)
Endocrine, nutritional, and metabolic diseases	22	14 (63.6%)	5 (35.7%)
Mental and behavioural disorders	12	10 (83.3%)	6 (60.0%)
Diseases of the nervous system	14	6 (42.9%)	4 (66.7%)
Diseases of the circulatory system	36	22 (61.1%)	6 (27.3%)
Diseases of the respiratory system	21	17 (80.9%)	5 (29.4%)
Diseases of the digestive system	27	12 (44.4%)	3 (25.0%)
Diseases of the skin and subcutaneous tissue	8	6 (75.0%)	1 (16.7%)
Diseases of the musculoskeletal system and connective tissue	15	10 (66.7%)	3 (30.0%)
Diseases of the genitourinary system	28	16 (57.1%)	5 (31.2%)
Symptoms, signs, and abnormal clinical and laboratory findings, not elsewhere classified	8	6 (75.0%)	1 (16.7%)
Others	9	5 (55.5%)	1 (20.0%)
Total	227	135 (59.5%)	42 (31.1%)

**Table 4:** The primary outcome reporting percentage with sample size.

Sample size	No. of RCTs	No. of RCTs that reported primary outcome	No. of RCTs that adopted multiple primary outcomes
Small	52	24 (46.1%)	9 (37.5%)
Medium	118	59 (50.0%)	17 (28.8%)
Large	57	52 (91.2%)	16 (30.8%)
Total	227	135 (59.5%)	42 (31.1%)
*P* value		0.000	0.739

## Data Availability

The data used to support the study are available from Professor Jing Hu (hujingebm@163.com).

## Supplementary Materials

Supplementary Materials PRISMA 2009 checklist. PRISMA 2009 flow diagram. MEDLINE (OVID) search strategy.
