# Supplementary figures and images for: The Use of Multiple Primary Outcomes in Randomized Controlled Trials of Chinese Herbal Medicine
Source: Evid Based Complement Alternat Med. 2021 Apr 21;2021:9975351. doi: 10.1155/2021/9975351 (PMC8081594; doi:10.1155/2021/9975351)

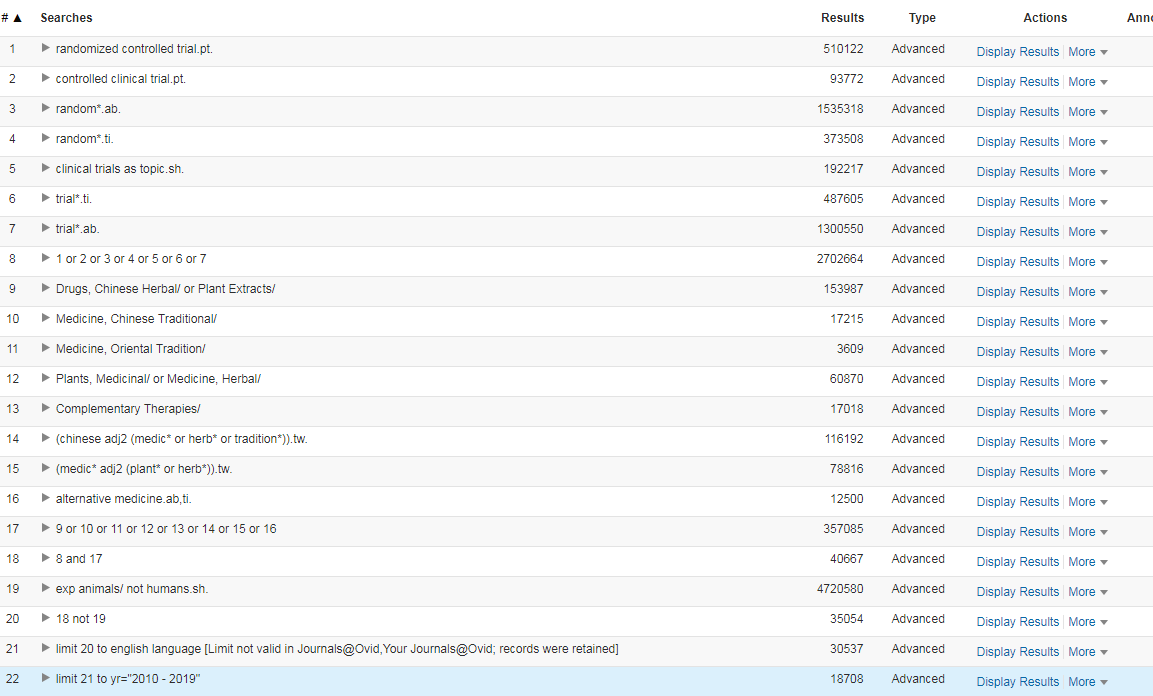

Supplement: Supplementary Materials — PRISMA 2009 checklist. PRISMA 2009 flow diagram. MEDLINE (OVID) search strategy. [file 9975351.f1.zip › 9975351.f1/MEDLINE (OVID) search strategy.docx]
