# Supplementary material for: The Use of Multiple Primary Outcomes in Randomized Controlled Trials of Chinese Herbal Medicine
Source: Evid Based Complement Alternat Med. 2021 Apr 21;2021:9975351. doi: 10.1155/2021/9975351 (PMC8081594; doi:10.1155/2021/9975351)
Supplement: Supplementary Materials — PRISMA 2009 checklist. PRISMA 2009 flow diagram. MEDLINE (OVID) search strategy. [file 9975351.f1.zip › 9975351.f1/PRISMA 2009 flow diagram.doc]

**PRISMA 2009 Flow Diagram**

**Screening**

**Included**

**Eligibility**

**Identification**

Records identified through database searching
(n = 39,116 )

Additional records identified through other sources
(n = 0 )

Records after duplicates removed
(n = 7,824 )

Records screened
(n = 7,824 )

Records excluded
(n = 7,349 )

Full-text articles assessed for eligibility
(n = 475 )

Full-text articles excluded, with reasons
(n =248	)

Studies included in qualitative synthesis
(n = 227 )

Studies included in quantitative synthesis (meta-analysis)
(n = 227 )
